# Chess classes and executive function skills in 5–6 years old children: evidence from cross-sectional study

**DOI:** 10.3389/fpsyg.2025.1564963

**Published:** 2025-07-21

**Authors:** Anastasia Yakushina, Elena Chichinina, Aleksandra Dolgikh

**Affiliations:** ^1^Federal Scientific Center of Psychological and Multidisciplinary Research (FSC PMR), Moscow, Russia; ^2^Faculty of Psychology, Lomonosov Moscow State University, Moscow, Russia

**Keywords:** executive function skills, cognitive development, chess classes, extracurricular activities, preschool children

## Abstract

The aim of this study was to compare executive function skills in preschool children who were engaged in chess classes with those who were not. The participants were 88 typically developing 5–6-year-old children. There were two groups with 30 boys and 14 girls in each group: a group of chess players and a group of non-chess players. The results indicated that children who participated in chess classes exhibited significantly higher visuospatial working memory scores compared to their non-chess playing counterparts (U = 731, *p* = 0.05). Moreover, the analysis revealed no significant differences in the characteristics of extracurricular activities undertaken by the two groups. Consequently, the enhanced visuospatial working memory levels observed in the chess-playing group may be due to their participation in chess classes. Taken together, these findings suggest that chess classes may represent a viable method for enhancing visuospatial working memory in preschool-aged children.

## 1 Introduction

Chess has been proven to be a mentally demanding activity that requires players to possess a range of cognitive skills such as critical thinking and strategic planning (Burgoyne et al., 2016). It has been demonstrated that playing chess can contribute to the development of self-discipline and the improvement of intellectual competences (Gobet, 2018). The game demands players to swiftly comprehend the gist of a given position and identify effective moves. These actions require the chess players to plan their own activity and anticipate potential moves of their opponents. Due to this fact the game involves important mental qualities such as visual imagination, memory, quickness of perception and tactical abilities (Gobet, 2018; Vasyukova and Mitina, 2024).

The popularity of chess among children is increasing annually (Sadkovkin, 2023). According to the World Chess Federation (FIDE),^1^ the number of children worldwide engaged in chess exceeds 25 million. In over 30 countries, chess classes are included in the recommendations for the development of preschool and school-age children (Hong and Bart, 2007; Joseph et al., 2016; Sala et al., 2017). Furthermore, the onset of children’s participation in chess classes has been documented to occur at around the age of 5–6 years (Glukhova, 2022; Sala et al., 2017).

Regular chess classes for preschool and school-age children are associated with the development of intelligence, visuospatial abilities, planning, and mathematical abilities (Burgoyne et al., 2016; Gao et al., 2019; Sigirtmac, 2012). In this context, the study of the relationship between cognitive development and chess classes in children has become relevant in recent years. It is further suggested that chess classes for preschoolers may serve as a viable method for enhancing executive function skills (cognitive skills that provide a group of target problem-solving and adaptive behaviour in new situations).

According to the Miyake model, there are the following main executive function skills (EF skills): (1) working memory (verbal and visuospatial) facilitates the retention and utilisation of information to solve current problems; (2) inhibition enables the suppression of dominant responses in favour of tasks needed; (3) cognitive flexibility facilitates the transition from one rule/condition to another (Miyake et al., 2000; Diamond, 2013).

The preschool period has been identified as a particularly sensitive period for the development of all EF skills (Silva et al., 2022; Bukhalenkova et al., 2023; Zukerman and Obukhova, 2024). Between the ages of 3 and 6, the brain of a child is highly plastic (Tierney and Nelson, 2009). The level of EF skills in preschool has been shown to be significantly correlated with various factors, including learning motivation, social skills and academic success at school (Cortés Pascual et al., 2019; Bubnovskaia et al., 2024; Robson et al., 2020).

EF skills in preschool age are influenced by a number of factors, including the quality of parents-child interaction (Valcan et al., 2018), the preschool educational programme (Veraksa et al., 2019; Gavrilova et al., 2024), the participation in play (Veraksa et al., 2024; Doebel and Lillard, 2023; Gashaj et al., 2021), screen time (McNeill et al., 2019) and involvement in extracurricular activities, for example, music classes (Chen et al., 2022; Dolgikh et al., 2022), dance classes (Rudd et al., 2021), sports (Chang et al., 2012), foreign language classes (Yuile and Sabbagh, 2021). Recent research has indicated that in order for any organised activity to contribute to the development of EF skills, it must meet a series of requirements (Diamond and Ling, 2016; Bergman Nutley et al., 2008). Firstly, the development of EF skills depends on the duration, frequency and regularity of classes (Diamond and Ling, 2016). Secondly, the tasks assigned to children during classes should present the “zone of proximal development” (Vygotsky, 2012). Thirdly, activities should support child motivation, promote self-confidence and social competence (Howie et al., 2010; Bang et al., 2020). In this regard, chess classes represent a potential extracurricular activity that may positively impact EF skills in preschoolers due to compliance with the criteria described above.

Research has shown that children with advanced EF skills process information faster, prevent irrelevant information more effectively, and correct their own errors more quickly. Chess also involves reasoning iteratively about the opponent’s potential intentional choices. Neurologically, chess practice has been shown to involve an increased activation of the prefrontal cortex associated with EF skills and visual perspective taking (Powell et al., 2017; Atherton et al., 2003). A growing body of evidence indicates a positive relationship between chess classes and EF skills (Unterrainer et al., 2006; Addarii et al., 2022; Khosrorad et al., 2014). Investigations have revealed that children who play chess have higher scores in cognitive flexibility, planning and inhibitory control compared to non-chess players.

It is important to acknowledge that the presented studies examine the role of chess classes in the development of executive function skills in schoolchildren. Nevertheless, the most sensitive period for the development of EF skills is preschool age (Monette et al., 2015; Scionti and Marzocchi, 2021). In this regard, it is important to consider the influence of chess classes on EF skills specifically in preschool age. Additionally, most studies do not indicate regularity of chess classes attendance and participation in other extracurricular activities. Children aged 5–6 years often start participating in extracurricular activities that contribute to EF skills development, including chess classes.

## 2 Current study

The aim of this study was to compare EF skills in preschool children aged 5–6 years who were chess players and non-chess players. The age group was selected for two primary reasons. Firstly, it is widely acknowledged that from 5 to 6 years of age, EF skills develop most intensively (Garon et al., 2008). Secondly, it is plausible that children may start attending chess classes at this age. The present study’s sample comprised children who had attended chess classes for a minimum period of 6 months. Given that the duration of class attendance in months, the duration of each class in minutes, and the frequency have been shown to be associated with EF skills outcomes, these indicators were also taken into account (Diamond and Ling, 2016). The novelty of this research lies in the fact that all core EF skills (working memory, inhibition, and cognitive flexibility) were studied while in most research involving preschoolers only specific EF skills were studied (Grau-Pérez and Moreira, 2017; Khosrorad et al., 2014). In order to achieve the aim of the study, we posed the following research question: How do EF skills differ between 5 and 6-year-old children attending extracurricular chess classes and children not attending extracurricular chess classes?

## 3 Materials and methods

### 3.1 Procedure

The current study took place between March and April 2024. The study was organized as follows. First, an online questionnaire was administered to caregivers to identify children who had attended extracurricular chess classes at the time of data collection for at least 6 months at various chess schools and clubs; these children formed the so-called “chess player group.” A group of children who did not attend chess classes but was similar to the “chess players” group in sociodemographic respects, was also formed. Both groups then participated in an EF skills assessment. Prior to the data collection, approval to conduct the study was obtained from the kindergartens’ principals and written informed consent was obtained from participants’ caregivers.

Caregivers received links to the questionnaire in kindergarten parent chat. The data from the questionnaire revealed that 44 children (14 girls and 30 boys) met the inclusion criteria and were thus included in the group of chess players. The inclusion criterion for the group of chess players was participation in extracurricular chess classes for a minimum period of 6 months. It was observed that the children in this group engaged in a variety of extracurricular activities beyond their participation in extracurricular chess classes. The inclusion criteria for the group of non-chess players were as follows: no participation in chess classes and participation in more than one type of extracurricular activity (because in the group of chess players, children participated in at least two types of extracurricular activities including chess). Based on the data from the questionnaire, 366 children met these criteria. To form the group of non-chess players of 44 children, simple random sampling (Noor et al., 2022) was used separately for boys and girls. This online questionnaire was also used to elaborate on sociodemographic data.

Then in both groups, EF skills assessment was conducted by professional psychologists indoors at the kindergarten. The EF skills assessment was carried out in the morning, between 8 and 11 am. All the EF skills tests were performed during individual meetings with each child (lasting about 20 min) in a quiet room in the kindergartens. The tasks were given to all children in the same order: visuospatial working memory assessment, verbal working memory assessment, inhibitory control assessment, cognitive flexibility assessment. Standardized verbal instructions were given to all the children.

This study and its consent procedures were conducted in accordance with the Declaration of Helsinki and approved by the Ethics Committee of the Federal Scientific Center of Psychological and Multidisciplinary Research (the approval No. 3 dated 15 February 2024).

### 3.2 Measures

A questionnaire was designed for caregivers of preschool-aged children in order to investigate the participation of preschoolers in extracurricular activities, as well as to gather sociodemographic data. Caregivers were asked to indicate extracurricular activities in which their child was engaged, including sports, dance, music, school preparation, drawing, foreign language, chess, and any other activities specified by caregivers. For each extracurricular activity type, caregivers were asked to specify the frequency of classes per week, the duration of one class in minutes, and the duration of participation in months. Furthermore, caregivers were asked to provide the number of years of their education and to select the most appropriate option to describe their family income level (‘below average’, ‘average’, ‘above average’ and an option to provide an alternative response if necessary). Data on maternal education and family income was used to describe the sample.

The NEPSY-II subtests validated for the Russian population were employed to measure EF skills (Veraksa et al., 2020; Korkman et al., 2007). The NEPSY-II ‘Naming and Inhibition’ subtest was used to assess inhibitory control. This subtest specifically focuses on evaluating the speed of information processing and the ability to inhibit impulsive reactions. The first task is to identify the form (circle or square) as quickly as possible. Involves inverting the former task, such that if a square is demonstrated, he/she is supposed to say ‘circle’ and vice versa. The NEPSY-II ‘Sentences Repetition’ subtest (Korkman et al., 2007) was used to assess verbal working memory. The child is presented with 17 sentences of increasing complexity and length, one at a time, and is asked to repeat them. The NEPSY-II ‘Memory for Designs’ subtest was employed to evaluate visuospatial working memory. The child is shown a grid with 4 to 8 designs on a page for 10 s. The child is asked to select the designs from a set of cards and place them on a grid in the same location as previously shown. In the set of cards, there are target designs and distracter designs which look very similar to the target designs. Content score assesses the child’s ability to recall a design’s details. Spatial score assesses the child’s ability to recall the location of a design. Bonus score reflects the child’s ability to recall both a design’s details and the location of a design simultaneously. Total score (the sum of content score, spatial score and bonus score) reflects the child’s visuospatial working memory in general. The ‘The Dimensional Change Card Sort’ test was used to assess cognitive flexibility (Zelazo, 2006). This technique consists of three tasks for sorting cards. Firstly, the child is instructed to sort the cards by color, then by shape, and eventually, follow a complex rule: if a card has a frame, it has to be sorted by color, and if there is no frame, by shape.

### 3.3 Participants

The study’s participants comprised 88 typically developing 5–6-year-old children. The participants were divided into two groups with 30 boys and 14 girls in each group. The groups were categorised based on their engagement with extracurricular chess classes, with one group comprising chess players, and the other comprising non-chess players. The children in the former group had participated in chess classes for a minimum period of 6 months and engaged in other extracurricular activities, except for chess classes. In contrast, the group of non-chess players did not participate in any chess-related activities, although they did engage in a range of other extracurricular activities. Children in the group of non-chess players did not have any experience of chess classes attendance. Except for extracurricular activities, both groups participated in the same classes according to the general kindergarten programme, which included: sport classes (3 times a week), music classes (2 times a week), drawing classes (1 time a week), crafts classes (1 time a week), math classes (2 times a week), grammar classes (2 times a week), science classes (1 time a week; Veraksa et al., 2019).

A comparison of the average age of chess players and non-chess players in the sample revealed no significant difference: 69.9 ± 4.11 months and 68.3 ± 5.61 months, respectively (U = 865, *p* = 0.18). Children in both groups were primarily from families with medium socioeconomic status. In the group of chess players, caregivers had an average period of education 16.2 ± 1.02 years and in the group of non-chess players 16.2 ± 0.99 years (U = 1,002, *p* = 0.75). In both groups, about 75% children were from families with average income level.

### 3.4 Data analysis

In the study, EF skills and the features of participation in extracurricular activities were compared between two independent groups (the group of chess players and the group of non-chess players). Descriptive statistics were conducted presenting continuous variables as median, IQR, range (minimum and maximum) and categorical data as %. The normality of the distribution was examined with the Shapiro–Wilk test. Since the data were not normally distributed, non-parametric tests were used.

First, in order to exclude the fact that the difference between the groups in EF skills was due to participation in other extracurricular activities, the groups were compared according to the features of the participation in extracurricular activities. Mann–Whitney-test (U) was used to assess differences between the group of chess players and the group of non-chess players in number of extracurricular activities child participates in, average weekly frequency of extracurricular activities, an average class duration, and period of participation in extracurricular activities in months. Chi-square (*χ*^2^) test was used to assess the difference between the group of chess players and the group of non-chess players in the number of children participating in each type of extracurricular activities. Second, Mann–Whitney-test (U) was used to assess differences in the EF skills measures between the group of chess players and the group of non-chess players.

Rank biserial correlation (r_b_) was used to calculate effect size for Mann–Whitney-test (U). Interpretation of rank biserial correlation was as follows: r_b_ < 0.10 represented very small effect, 0.10 < r_b_ < 0.29 represented small effect,0.30 < r_b_ < 0.49 represented moderate effect, and r_b_ ≥ 0.5 large effect (Funder and Ozer, 2019). Cramer’s V was used to calculate effect size for Chi-square (*χ*^2^) test. Interpretation of Cramer’s V for Chi-square (*χ*^2^) test with one degree of freedom was as follows: 0.10 < V < 0.30 represented small effect, 0.30 < V < 0.49 moderate effect, V > 0.50 large effect (Funder and Ozer, 2019). Statistical significance was set at *p* < 0.05. All statistical analyses were conducted using Jamovi version 2.0.0.0.

## 4 Results

### 4.1 Descriptive statistics for children’s participation in extracurricular activities and differences between the group of chess players and the group of non-chess players in extracurricular activities participation

Table 1 reports descriptive statistics for features of participation in extracurricular activities in the group of chess players and in the group of non-chess players. In the group of chess players, children attended chess classes on average of 2 times a week; each chess class lasted approximately 40 min and at the time of data collection the average period of attendance was 12 months.

**Table 1:** Descriptive statistics for children’s participation in extracurricular activities and differences between the group of chess players and the group of non-chess players in extracurricular activities participation.

Description of extracurricular activities	Group of chess players			Group of non-chess players			Mann–Whitney test		
	Median	IQR	Min; Max	Median	IQR	Min; Max	U	*p*	*r_b_*
Number of extracurricular activities a child participates in	3	2	1; 6	3	2	1; 5	898	0.26	0.13
Frequency of an extracurricular activity, times per week, on average	2	0.00	1; 3	2	0.25	1; 4	820	0.06	0.19
A class duration, minutes, on average	48	21.5	30; 120	50	18.5	20; 97	934	0.53	0.08
Period of participation in an extracurricular activity, months, on average	16	7	6; 24	18	12	6; 45	822	0.23	0.15
Frequency of an extracurricular chess classes, times per week, on average	2	1	1; 3						
A chess class duration, minutes, on average	40	30	10; 70						
Period of participation in extracurricular chess classes, months, on average	12	1.5	6; 24						

Type of extracurricular classes	Group of chess players	Group of non-chess players	Chi-square test		
	%	%	*χ* ^2^	*p*	*V*
Sport classes	69.6	82.2	1.99	0.16	0.15
Dance classes	26.1	40.0	1.99	0.16	0.16
Music classes	23.5	11.8	1.62	0.20	0.15
School preparation classes	52.2	64.4	1.41	0.24	0.12
Drawing classes	30.4	59.6	6.90	0.01	0.28
Foreign language classes	35.3	52.9	2.15	0.14	0.18
Robotics classes	2.9	2.9	0.00	1.00	0.00
Acting classes	2.9	2.9	0.00	1.00	0.00
Other extracurricular classes	5.9	8.8	0.22	0.64	0.06

Table 1 also reports the differences between the group of chess players and the group of non-chess players in extracurricular activities participation. The study revealed no significant disparities between the two groups in terms of participation in extracurricular activities. On average, children participated in three types of classes. Participation in these classes occurred, on average, twice per week. The duration of each class was approximately 50 min on average. The duration of extracurricular activities’ attendance was found to be at the time of data collection approximately 16–18 months (about 1.5 years) for both groups. The effect sizes of these results were negligible, with the average duration of one class exhibiting the smallest effect size. The study revealed no significant differences between the groups in terms of the percentage of children participating in sports, dance, music, school preparation, foreign language, robotics, acting and other extracurricular activities. However, a statistically significant difference was observed in the percentage of children participating in drawing classes, with a higher proportion of non-chess players engaging in this activity compared to chess players. The effect sizes of these results were negligible.

### 4.2 Descriptive statistics for EF skills and differences between the group of chess players and the group of non-chess players in EF skills

Table 2 reports the differences between the group of chess players and the group of non-chess players in EF skills. The group of chess players was found to be significantly superior to the group of non-chess players with visuospatial working memory abilities: the ability to encompass the recall of the location of a given design (spatial score), and the ability to recall both the location and the details simultaneously (bonus score). The effect sizes of these results ranged from small to moderate. However, visuospatial working memory ability to recall a design’s details (content score), inhibitory control, verbal working memory, and cognitive flexibility did not differ between the two groups. Effect sizes for the ability to recall which designs were shown for each trial and verbal working memory were found to be small, while inhibitory control and cognitive flexibility demonstrated very small effect sizes.

**Table 2:** Descriptive statistics for EF skills and differences between the group of chess players and the group of non-chess players in EF skills.

EF skills	Group of chess players			Group of non-chess players			Mann–Whitney test		
	Median	IQR	Min; Max	Median	IQR	Min; Max	*U*	*p*	*r_b_*
Inhibitory control	10	5.75	3; 18	10	5	6; 16	1,017	0.89	0.02
Verbal working memory	19.5	4	12; 29	19	5	0; 27	867	0.40	0.10
Visuospatial working memory, content score	37	10	13; 48	36	6.5	25; 48	871	0.42	0.10
Visuospatial working memory, spatial score	20	5	7; 24	17	6	11; 24	718	0.04	0.26
Visuospatial working memory, bonus score	17	18	0; 48	12	9	0; 48	669	0.01	0.31
Visuospatial working memory, total score	75.5	32.5	32; 120	66	21.3	39; 120	731	0.05	0.25
Cognitive flexibility	20	3	15; 24	21	4.5	10; 24	841	0.47	0.09

## 5 Discussion

The aim of this study was to compare EF skills in preschool children aged 5–6 years who were chess players and non-chess players. The results of the study demonstrated that children who participated in chess classes had higher visuospatial working memory scores in comparison to those who did not attend such classes. It is noteworthy that chess players had higher spatial scores (ability to recall the location of a design) and bonus scores (ability to recall the location and design’s details simultaneously) compared to non-chess players. However, no significant differences were found for the content scores (ability to recall a design’s details). The findings can be explained by the hypothesis that, in order to identify promising solutions in chess with speed, it is necessary to accurately memorise and imagine perceptual units that can be treated as wholes, and to anticipate potential actions (Jastrzembski et al., 2006). The success of these actions requires sufficiently constant retention and processing of visuospatial information.

We believe that learning and playing chess actively engage visuospatial working memory. It is important to emphasize that chess instruction from the very beginning of chess education involves extensive training of visuospatial memory. Starting from their first lessons, children learn to memorize the correct placement of chess pieces, associate symbolic notation (using letters and numbers) with actual board positions, and visualize proper piece movements (Kosteniuk and Kosteniuk, 2008; Gobet, 2018). For instance, a common introductory exercise requires children to set up the initial chessboard configuration accurately and swiftly (Kosteniuk and Kosteniuk, 2008). Another foundational task focuses on practicing standard piece maneuvers such as guiding a knight to capture a pawn in a set number of moves. These structured exercises ensure that budding chess players immediately engage with and process substantial visuospatial information.

In the current study, there were differences between the groups in ability to recall the location of a design (spatial score) but not in the ability to recall a design’s details (content score). We find the results obtained to be logical. As for content information, there are only six chess pieces (king, queen, rook, bishop, knight, and pawn) that the chess player needs to keep in visual spatial working memory. And as for spatial information, it is abundant during chess education and chess games.

Furthermore, chess practice and perspective-taking tasks involve the same brain areas. For instance, Powell et al. (2017) found out that when chess players estimate the opponent’s possible mental reasoning, the right temporoparietal junction becomes active. The activity of this structure is also important in distinguishing the first- or third-person perspective (Lawrence et al., 2006). In addition, Bukach et al. (2006) have found that the fusiform gyrus activates in both chess playing and visuospatial capture tasks (Bukach et al., 2006).

The result of our study is congruent with those reported in the research by Sigirtmac (2012). The research was conducted on a sample of 100 children aged 6 years, 50 of them being chess players. The study revealed that chess players exhibited a higher level of development in their visuospatial abilities when compared to non-chess players. The study’s findings suggest that children who practice chess have a better understanding of what visuospatial concepts mean (e.g., forward–backward, between–next to, in front–behind, diagonal, far–near, corner). Additionally, the children exhibited an ability to correctly place objects according to the required direction (Sigirtmac, 2012). A similar finding was reported in a study conducted by Gao et al. (2019). which involves a sample of 30 children aged 11–12 years. The study revealed that children who engaged in daily after-school chess practice for a period of 5 years, dedicating 2 h to the game, demonstrated superior proficiency in ascertaining whether a visual image displayed on a screen corresponded to a specific visuospatial perspective, such as the first- or third-person perspective. This proficiency was notably superior to that observed in non-chess players (Gao et al., 2019).

Furthermore, the “Memory for Design” subtest used in the current study for visuospatial working memory assessment closely resembles the cognitive exercises involved in chess training. In both tasks, children should memorize and recall the positions of objects arranged on a grid-like structure, mirroring the spatial organization of a chessboard. We assume that also in this regard, children who were chess players were able to perform better on the task compared to non-chess players.

In the course of our research, we conducted a study of two distinct groups: one comprising chess players and other comprising non-chess players. The children in the study attended a variety of extracurricular activities (in average three different types). We assumed that differences in EF skills between the groups are due to differences in types of these activities, the frequency or duration of classes. As research has demonstrated that extracurricular activities such as sports, dance and music classes influence EF skills, and the total number of extracurricular activities a child attends may affect EF skills (Contreras-Osorio et al., 2021; Rodriguez-Gomez and Talero-Gutiérrez, 2022; Veraksa et al., 2023). However, in our study no differences in the features of extracurricular activities participation were found. The only difference we have indicated is the number of children attending drawing classes: which was higher in the group of non-chess players. Given the established correlation between drawing classes and visuospatial working memory (Perdreau and Cavanagh, 2015), we can assume that the higher level of visuospatial working memory observed in chess players was associated with chess classes, rather than to their engagement in other extracurricular activities.

No differences in verbal memory, cognitive flexibility and inhibitory control were found in our study between chess players and non-chess players children. In our opinion, this result illustrates the possibility of developing indicated EF skills effectively through other extracurricular activities, such as music, sports or dances (Bayanova et al., 2024; Contreras-Osorio et al., 2022).

## 6 Limitations

The main limitations of our preliminary study are the relatively modest number of study participants and the correlational design. To understand how chess classes affect the EF skills development, experimental study is needed. In addition, in our study, there is a lack of information on baseline differences in EF skills between children from the chess players group and from the non-chess players group. Also, the limitation is that this study did not specify whether the children voluntarily participated in extracurricular chess activities. An additional limitation of this study pertains to the absence of data regarding home chess-related practices among children in both groups, as well as insights into chess-related conceptual understanding among children from the non-chess group. A questionnaire for caregivers should be developed to examine children’s motivation to practice chess, parents’ attitudes towards chess classes, and home chess-related practices. Furthermore, the study did not take into account family factors that may influence EF skills, such as the quality of parent–child interactions, the home environment and family joint activities.

## 7 Conclusion

This study sets out to determine the potential impact of chess classes on the EF skills in preschool-aged children. The results of the study indicated that children who attend extracurricular chess classes have more developed visuospatial working memory compared to children who do not attend such classes. Based on the results of the study, we assume that chess classes can potentially be catalysts for visuospatial working memory development. The results obtained are of practical significance for teachers and caregivers: chess classes can be an effective way of improving visuospatial working memory in young children. Schools and kindergartens could incorporate short, game-based chess sessions, teachers could learn the basic chess rules to guide children more effectively. Parents could introduce children to chess through fun activities such as mobile apps, family games, or chess clubs. To achieve optimal results, chess could be combined with other exercises that improve memory and spatial reasoning. Education authorities and parent communities could promote chess accessibility, particularly for children from diverse socioeconomic backgrounds.

## Data Availability

The raw data supporting the conclusions of this article will be made available by the authors, without undue reservation.

## Footnotes

^1^ https://www.fide.com
